# Supplementary material for: GWAS of Reproductive Traits in Large White Pigs on Chip and Imputed Whole-Genome Sequencing Data
Source: Int J Mol Sci. 2022 Nov 1;23(21):13338. doi: 10.3390/ijms232113338 (PMC9656588; doi:10.3390/ijms232113338)
Supplement: Supplementary file 1 [file ijms-23-13338-s001.zip › Supplementary figure.pdf]

Figure S1

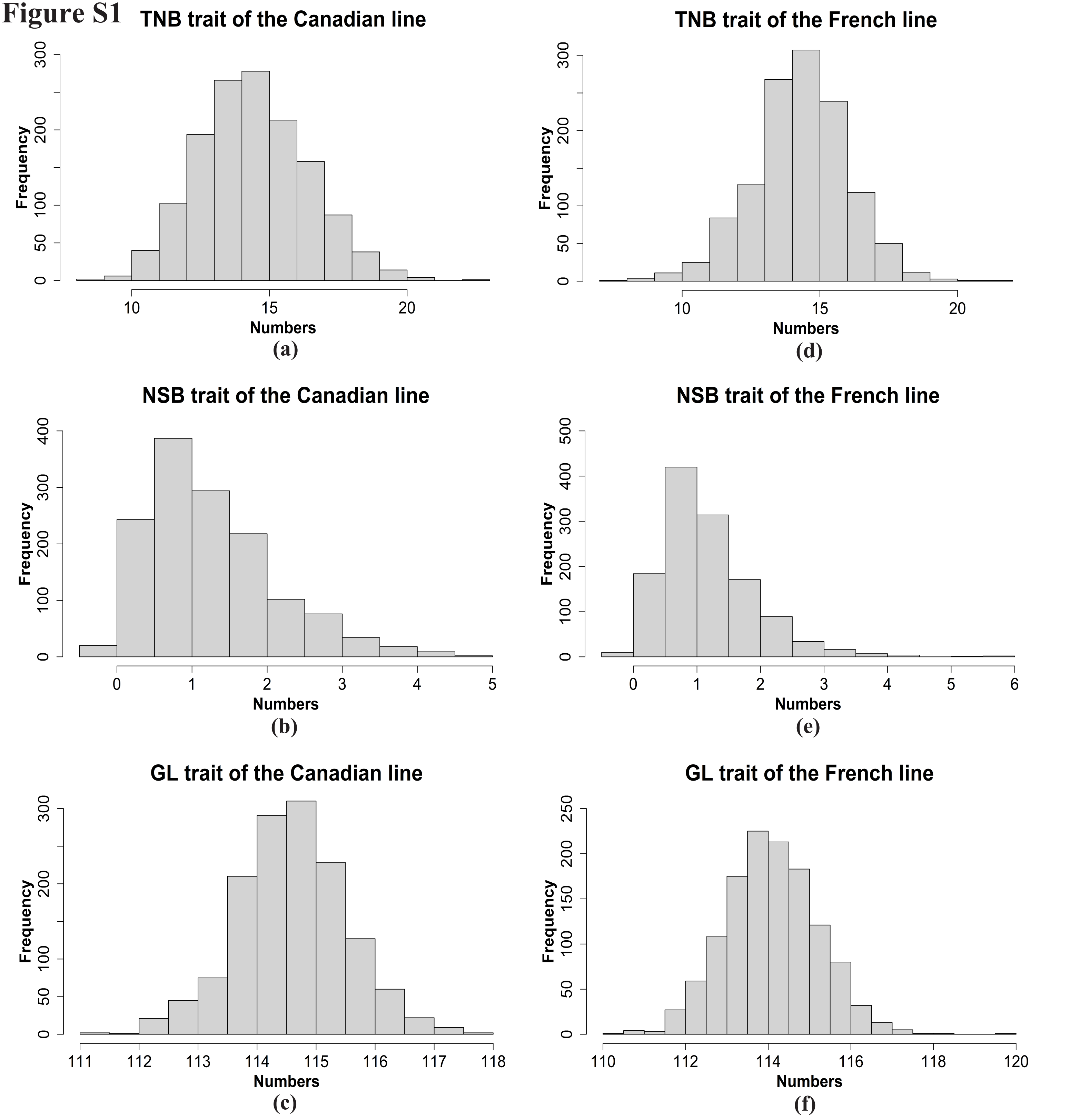
TNB trait of the Canadian line
Frequency
0
50
100
200
300
10
15
20
Numbers
(a)
TNB trait of the French line
Frequency
0
50
100
200
300
10
15
20
Numbers
(d)
NSB trait of the Canadian line
Frequency
0
100
200
300
400
0
1
2
3
4
5
Numbers
(b)
NSB trait of the French line
Frequency
0
100
200
300
400
500
0
1
2
3
4
5
6
Numbers
(e)
GL trait of the Canadian line
Frequency
0
50
100
200
300
111
112
113
114
115
116
117
118
Numbers
(c)
GL trait of the French line
Frequency
0
50
100
150
200
250
110
112
114
116
118
120
Numbers
(f)

Figure S2

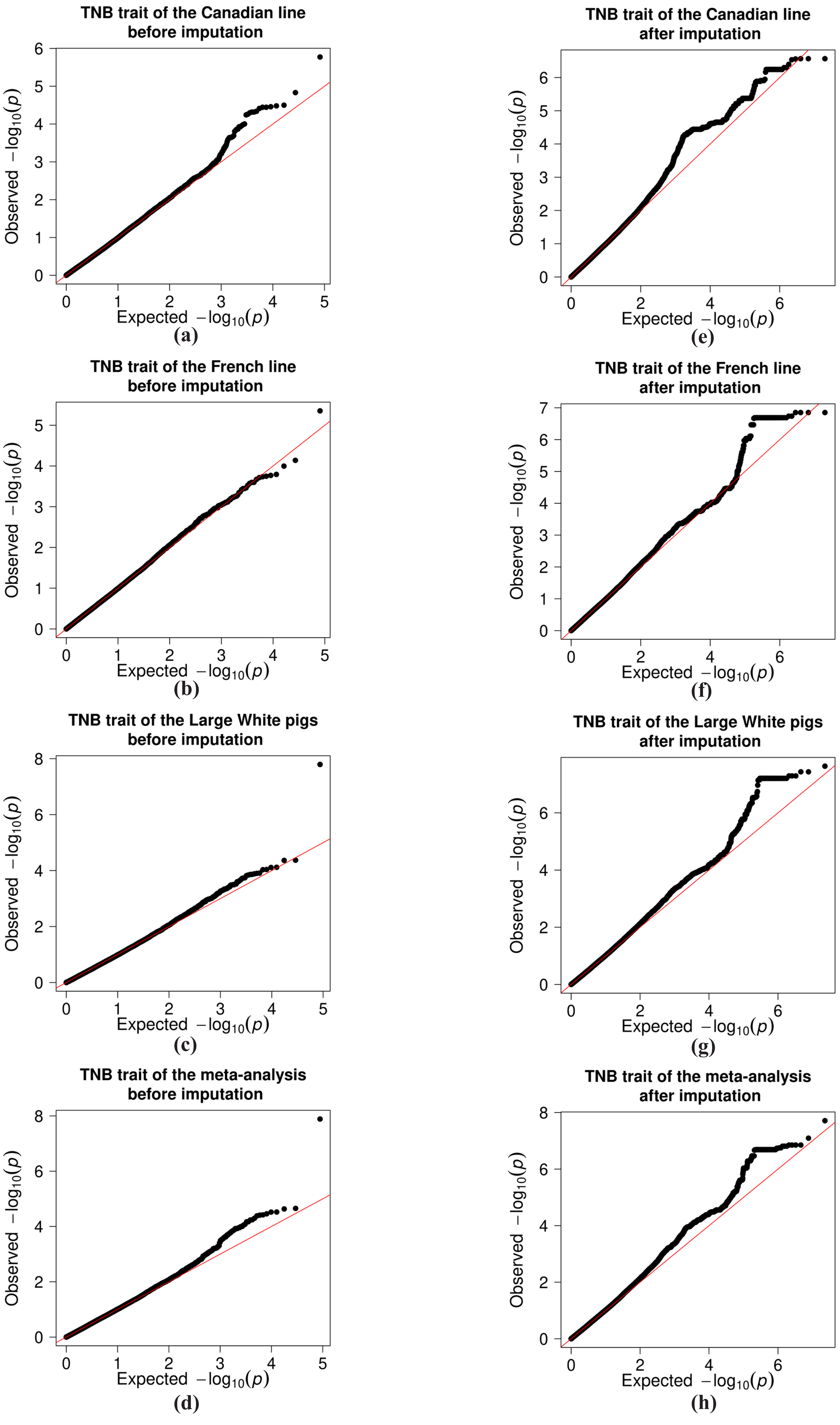
TNB trait of the Canadian line
before imputation
Observed −log10(p)
Expected −log10(p)
(a)
TNB trait of the Canadian line
after imputation
Observed −log10(p)
Expected −log10(p)
(e)
TNB trait of the French line
before imputation
Observed −log10(p)
Expected −log10(p)
(b)
TNB trait of the French line
after imputation
Observed −log10(p)
Expected −log10(p)
(f)
TNB trait of the Large White pigs
before imputation
Observed −log10(p)
Expected −log10(p)
(c)
TNB trait of the Large White pigs
after imputation
Observed −log10(p)
Expected −log10(p)
(g)
TNB trait of the meta-analysis
before imputation
Observed −log10(p)
Expected −log10(p)
(d)
TNB trait of the meta-analysis
after imputation
Observed −log10(p)
Expected −log10(p)
(h)

Figure S3

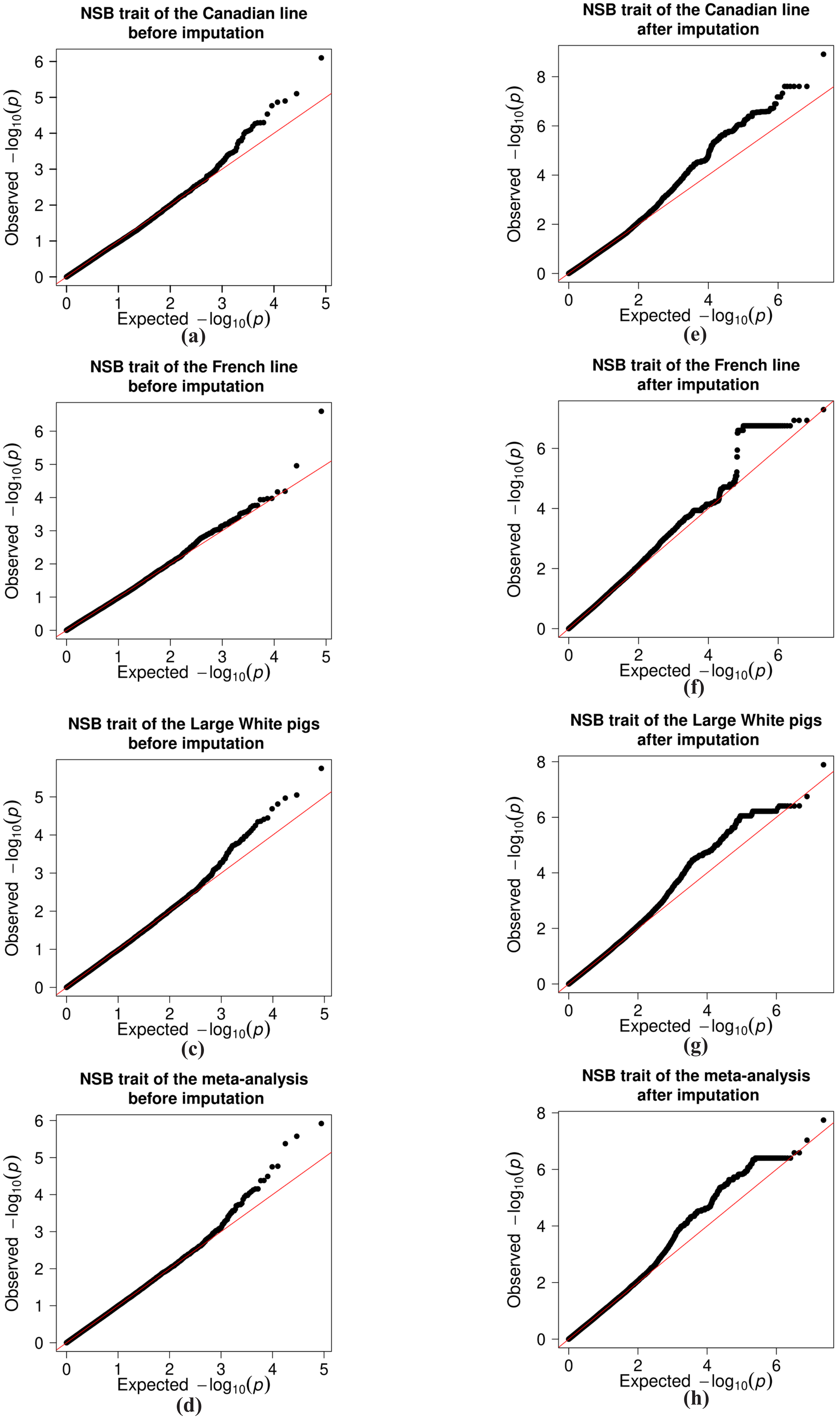
NSB trait of the Canadian line before imputation
Observed −log10(p)
Expected −log10(p)
(a)
NSB trait of the Canadian line after imputation
Observed −log10(p)
Expected −log10(p)
(e)
NSB trait of the French line before imputation
Observed −log10(p)
Expected −log10(p)
(b)
NSB trait of the French line after imputation
Observed −log10(p)
Expected −log10(p)
(f)
NSB trait of the Large White pigs before imputation
Observed −log10(p)
Expected −log10(p)
(c)
NSB trait of the Large White pigs after imputation
Observed −log10(p)
Expected −log10(p)
(g)
NSB trait of the meta-analysis before imputation
Observed −log10(p)
Expected −log10(p)
(d)
NSB trait of the meta-analysis after imputation
Observed −log10(p)
Expected −log10(p)
(h)

Figure S4

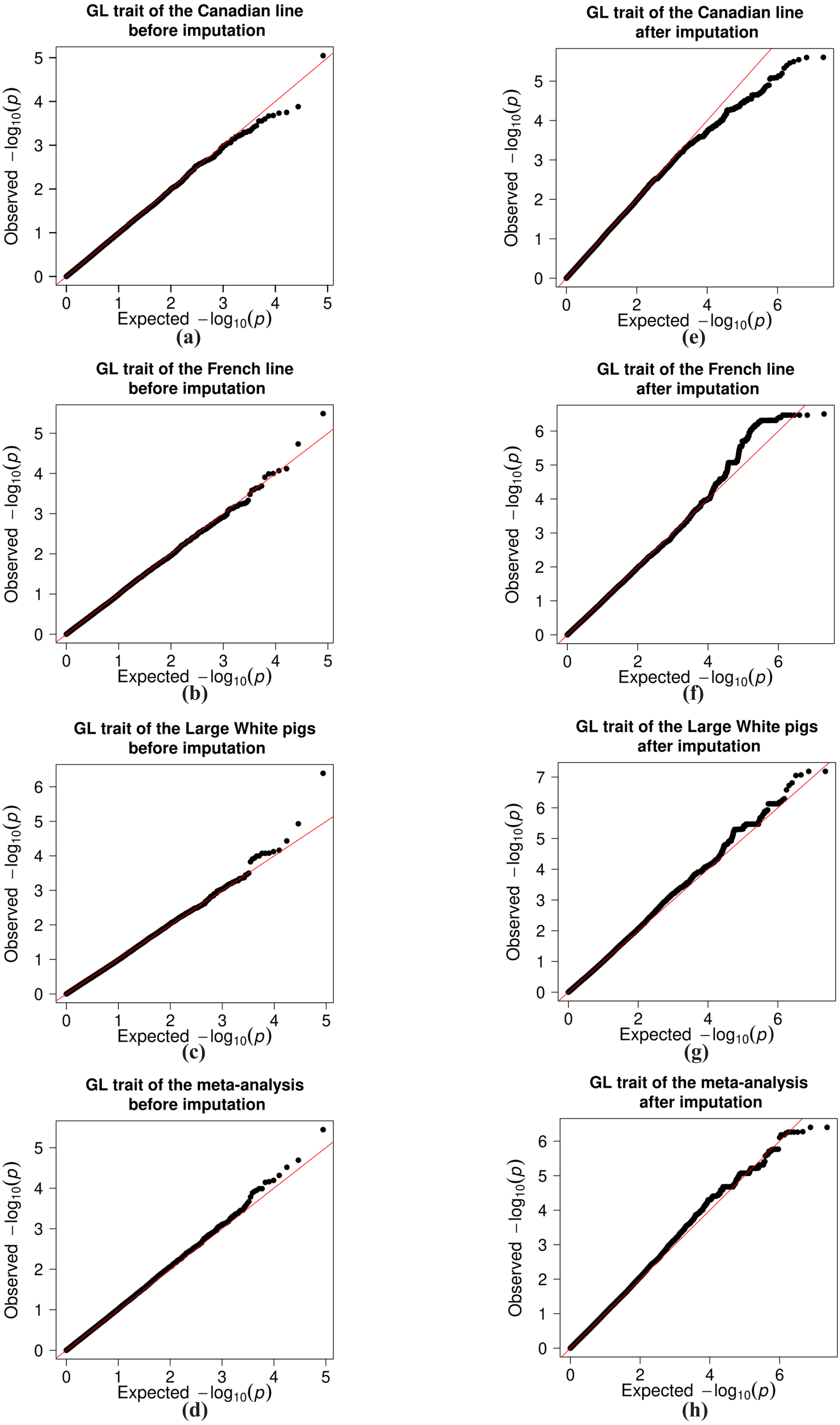
GL trait of the Canadian line
before imputation
Observed $-\log_{10}(p)$
Expected $-\log_{10}(p)$
(a)
GL trait of the Canadian line
after imputation
Observed $-\log_{10}(p)$
Expected $-\log_{10}(p)$
(e)
GL trait of the French line
before imputation
Observed $-\log_{10}(p)$
Expected $-\log_{10}(p)$
(b)
GL trait of the French line
after imputation
Observed $-\log_{10}(p)$
Expected $-\log_{10}(p)$
(f)
GL trait of the Large White pigs
before imputation
Observed $-\log_{10}(p)$
Expected $-\log_{10}(p)$
(c)
GL trait of the Large White pigs
after imputation
Observed $-\log_{10}(p)$
Expected $-\log_{10}(p)$
(g)
GL trait of the meta-analysis
before imputation
Observed $-\log_{10}(p)$
Expected $-\log_{10}(p)$
(d)
GL trait of the meta-analysis
after imputation
Observed $-\log_{10}(p)$
Expected $-\log_{10}(p)$
(h)
