# Supplementary material for: GWAS of Reproductive Traits in Large White Pigs on Chip and Imputed Whole-Genome Sequencing Data
Source: Int J Mol Sci. 2022 Nov 1;23(21):13338. doi: 10.3390/ijms232113338 (PMC9656588; doi:10.3390/ijms232113338)
Supplement: Supplementary file 1 [file ijms-23-13338-s001.zip › Supplementary table.pdf]

Supplementary Table S1. The number of SNPs before and after imputation, and the number of SNPs used for GWAS in the imputation-based WGS data after imputation quality control in each chromosome. The imputation accuracy R^2 before and after quality control in each chromosome.

Chr	Before imputation	After imputation			
		Before quality control		After quality control	
	N_SNPs	N_SNPs	R^2	N_SNPs	R^2
1	4,477	1,339,285	0.884	1,075,629	0.951
2	3,024	999,583	0.874	803,914	0.943
3	2,695	926,745	0.880	729,297	0.948
4	2,687	805,630	0.875	636,131	0.946
5	2,084	715,246	0.874	566,581	0.942
6	3,105	1,085,543	0.878	856,276	0.944
7	2,556	827,816	0.876	658,652	0.942
8	2,674	932,853	0.858	716,623	0.939
9	2,897	886,752	0.887	717,149	0.947
10	1,377	692,778	0.859	523,450	0.928
11	1,687	636,509	0.862	490,523	0.935
12	1,233	531,749	0.861	405,278	0.930
13	3,383	1,029,986	0.883	822,649	0.948
14	2,965	922,836	0.876	718,359	0.946
15	2,672	763,715	0.890	627,684	0.947
16	1,596	553,301	0.877	443,281	0.940
17	1,277	514,149	0.867	399,292	0.938
18	1,160	396,969	0.908	331,068	0.951

Chr, chromosome; N_SNPs, number of SNPs; R^2, imputation accuracy.

Supplementary Table S2. The genomic inflation factor (λ) for each GWAS using chip data in pigs.

Trait	λ (Canadian)	λ (French)	λ (Combined LW)	λ (Meta)
Total number born (TNB)	0.960	1.000	0.950	0.985
Number of stillborn (NSB)	0.957	1.006	0.968	1.003
Gestation length (GL)	0.973	0.978	0.989	1.024

Supplementary Table S3. The genomic inflation factor (λ) for each GWAS using imputed WGS data in pigs.

Trait	λ (Canadian)	λ (French)	λ (Combined LW)	λ (Meta)
Total number born (TNB)	0.969	0.987	0.977	0.993
Number of stillborn (NSB)	0.938	1.036	0.977	1.013
Gestation length (GL)	0.975	0.975	0.990	1.024
